# Idebenone Antagonizes P53-Mediated Neuronal Oxidative Stress Injury by Regulating CD38-SIRT3 Protein Level

**DOI:** 10.1007/s11064-024-04189-7

**Published:** 2024-06-12

**Authors:** Hao Xu, Ying Guo, Xiao-Jun Liu, Ying Liu, Shi Yin, Qi-Ying Bao, Ru Peng, Wei-Bo Tian, Ying-Yan Xia, Ling Gao, Jia-Mei Liu

**Affiliations:** 1 Department of Histology and Embryology, College of Basic Medical Sciences, Jilin University, Changchun, P.R. China; 2 China-Japan Union Hospital of Jilin University, Changchun, 130033 Jilin China; 3 Jiangsu Health Vocational College, Nanjing, P.R. China; 4 Jilin University, Changchun, P.R. China; 5 Bethune Second Clinical School of Medicine, Jilin University, Changchun, P.R. China; 6 Basic medical department of Changchun Medical College, Changchun, P.R. China

**Keywords:** Idebenone, H_2_O_2_, CD38, SIRT3, P53, Deacetylation

## Abstract

Idebenone, an antioxidant used in treating oxidative damage-related diseases, has unclear neuroprotective mechanisms. Oxidative stress affects cell and mitochondrial membranes, altering Adp-ribosyl cyclase (CD38) and Silent message regulator 3 (SIRT3) protein expression and possibly impacting SIRT3’s ability to deacetylate Tumor protein p53 (P53). This study explores the relationship between CD38, SIRT3, and P53 in H_2_O_2_-injured HT22 cells treated with Idebenone. Apoptosis was detected using flow cytometry and terminal deoxynucleotidyl transferase-mediated dUTP nick end labeling (TUNEL) staining after determining appropriate H_2_O_2_ and Idebenone concentrations.

In this study, Idebenone was found to reduce apoptosis and decrease P53 and Caspase3 expression in H_2_O_2_-injured HT22 cells by detecting apoptosis-related protein expression. Through bioinformatics methods, CD38 was identified as the target of Idebenone, and it further demonstrated that Idebenone decreased the expression of CD38 and increased the level of SIRT3. An increased NAD^+^/NADH ratio was detected, suggesting Idebenone induces SIRT3 expression and protects HT22 cells by decreasing apoptosis-related proteins. Knocking down SIRT3 downregulated acetylated P53 (P53Ac), indicating SIRT3’s importance in P53 deacetylation.

These results supported that CD38 was used as a target of Idebenone to up-regulate SIRT3 to deacetylate activated P53, thereby protecting HT22 cells from oxidative stress injury. Thus, Idebenone is a drug that may show great potential in protecting against reactive oxygen species (ROS) induced diseases such as Parkinson’s disease, and Alzheimer’s disease. And it might be able to compensate for some of the defects associated with CD38-related diseases.

**Supplementary Information:**

The online version contains supplementary material available at 10.1007/s11064-024-04189-7.

## Introduction

Oxidative stress injury is an imbalance state between oxidation and antioxidant effects in the body, often accompanied by mitochondrial dysfunction [1], in which the ability of mitochondria to scavenge reactive oxygen species (ROS) is reduced, leading to ROS accumulation [2]. ROS is the primary reason to neuron damage, eventually leading to various neurodegenerative diseases, such as ischemic stroke, Parkinson’s disease [3], Alzheimer’s disease [4], and amyotrophic lateral sclerosis.

Idebenone is an analog of short-chain coenzyme Q, a lipophilic electron carrier and an endogenous antioxidant found in all cell membranes which transfers electrons to the electron transport chain complex III [19] in mitochondrial intima. It ameliorates mitochondrial dysfunction [20] and protects cells by scavenging ROS [21]. Idebenone has been shown to up-regulate SIRT3 in vascular endothelial cells, activate the SIRT3-SOD2-MTROs pathway, and play a protective role in atherosclerotic blood vessels [22]. However, whether Idebenone can protect neurons by regulating oxidative stress, that is, the specific mechanism of its regulation of oxidative stress, is still unclear.

This study investigated the mechanism involved in the role of Idebenone on oxidative stress in H_2_O_2_-injured neuronal cells in vitro. It was verified that CD38 was one of the therapeutic targets of Idebenone. It was found that Idebenone inhibited the expression of CD38 and up-regulated SIRT3, which induced the deacetylation of P53 in mitochondria and ultimately reduced P53-mediated neuronal apoptosis. It provides better guidance for the clinical medication of Idebennone. In addition, Idebenone could be a potential drug for related diseases that target CD38 protein.

## Methods

### Drug Preparation and Cell Culture

Idebenone (Lot No. I0848) was purchased from TCI Chemical Industrial Development Co., LTD., Shanghai, China. Idebenone with purity > 98% was dissolved in Dimethyl Sulfoxide (DMSO) (purity > 99.9%, Beyotime Biotech Co., LTD, China) to make a solution of 20mM for in vitro experiment. Mouse hippocampal neuron cell line HT22 (purchased from ATCC, U.S.) was used to establish the model of neuronal oxidative stress injury. HT22 cells were cultured in Dulbecco’s Modified Eagle Medium (DMEM, Gibco, U.S.A.) containing 10% fetal bovine serum (Hyclone, U.S.A) and 1% penicillin/streptomycin (Dingguo Biotech Co, China) and were incubated at 37 °C in an atmosphere of 5% CO_2_. The cells were used when they reached a cellular confluence of ± 80–90%. Cell experiments were carried out after the second to fourth passage when cell viability was restored.

### Establishment of Oxidative Stress Cell Model HT22

Cells were cultured for 24 h in a 96-well plate. Hydrogen peroxide (H_2_O_2_) (Dingguo Biotech Co, China) respectively with concentrations of 0, 50, 100, 150, 200, 250, 300, 400 and 500µM were added to each 5 wells. After culturing for 6 h, cell morphology was observed under a microscope. Combining with results obtained from 3-(4,5-dimethylthiazol-2-yl)-2,5-diphenyltetrazolium bromide (MTT) assays, a dose-effect curve was drawn and the optimal concentration of H_2_O_2_ was selected for subsequent experiments.

### Establishment of Therapeutic Model of Idebenone in H_2_O_2_ Injuried Cells

HT22 cells were cultured in 96-well plates for 24 h to 60–70% confluence, and were pretreated respectively with 0, 10, 20, 25, 30, 40 and 50µM Idebenone for 2 h, and then treated with 250µM H_2_O_2_ for 6 h. Cell morphology was observed under a microscope (Model and Country). The dose-response curve was drawn combining with the results obtained from MTT assays, and an appropriate concentration of Idebenone was selected for subsequent experiments.

### Cell Viability and Cytotoxicity Assays

Cell activity was determined by MTT colorimetry. HT22 cells were grouped and seeded into 96-well plates, about 7500 cells per well. Idebenone-treated HT22 cells were incubated in complete DMEM medium containing 0.5 mg/mL MTT (Beyotime Biotech Co., LTD, China) for 4 h. Then 100 µL DMSO solution was added to dissolve the formazan generated in the cells. Ten minutes later, the optical density (OD) values were read by a Microplate Reader (BIOTEK, ELX-800, U.S.A.) at 570 nm. Lactate dehydrogenase (LDH; 10 µl) was added to the cells using an LDH cytotoxicity assay kit (C0016, Beyotime Institute of Biotechnology, China) according to the manufacturer’s protocol. Cytotoxicity was measured using the Enzyme-Linked Immunosorbent Assay (ELISA) reader (Beckman Coulter, Inc.) at a wavelength of 490 nm.

### Flow Cytometry

Flow cytometry assays were used to determine the death of cell induced by apoptosis. HT22 cells were inoculated in 6-well plates with 10^6 cells per well and cultured for 24 h. 20µM Idebenone was added to Idebenone pretreatment group 24 h. Later, 250µM H_2_O_2_ was added to Idebenone pretreatment group and to H_2_O_2_ injuried group 2 h later. These three groups of cells were digested 6 h later, then 195 µl binding solution, 5 µl Annexin V-FITC and 10 µl PI staining solution were added to each tube and mixed well. After incubation at room temperature for 25 min, the apoptosis of the cells was detected by using a Cytometer model. Flow cytometry analyses gating strategy is as followed. Early apoptosis: Annexin V-FITC single positive, Q3; late apoptosis: Annexin V-FITC and PI double positive, Q2; the percentage of apoptotic cells was measured as the sum of Q2 and Q3. According to the instructions of the reagent kit used in the experiment, each group tests 80,000 cells.

### TUNEL Staining

This method was used to detect cell apoptosis. HT22 cells were plated in a six-well plate and grouped as before. Then, 20µM Idebenone was added to the Idebenone treatment group and treated for 2 h. Then, 250µM H_2_O_2_ was added to the Idebenone pre-treatment group and H_2_O_2_ injuried group, respectively, and incubated for 6 h. After that,10 µl of Hochest33342 staining solution was added to each well, and incubated at room temperature in the dark for 10 min. Then,1 ml of PBS was added for washing. 1 ml of 4% paraformaldehyde was added to each well, and incubated at room temperature in the dark for 30 min. Then, 1 ml of 3% Triton-X was added to each well, and incubated at room temperature in the dark for 5 min. After that, 200 µl of TUNEL staining solution was added to each well, and incubated at 37℃ in the dark for 1 h. The cells were observed in search of apoptotic characteristics under an inverted fluorescence microscope (BX53, Japan).

### Target Prediction and Protein-Protein Interaction (PPI) Analysis of Idebenone

PubChem website (https://pubchem.ncbi.nlm.nih.gov/) was visited to analyze the 2D and 3D structure of Idebenone. Then, the 3D structure of Idebenone and PharmMapper website (http://www.lilab-ecust.cn/pharmmapper/) were consulted to predict the possible targets of Idebenone. For potential drug targets, the interactions between targets were explored using STRING database(https://cn.string-db.org), and then visualized using Cytoscape software (Cytoscape 3.8.0)).

### SiRNA Transfection

SIRT3 siRNA (mouse, GM-123507) Forward, 5’-GACCUUUGUAACAGCUACATT-3’; Reverse, 5’-UGUAGCUGUUACAAAGGUCTT-3’and control siRNA (GM-122,497) were purchased from GenePharma Co., LTD. (Suzhou, China), and transfection reagent (FT301) was purchased from TransGen Biotechnology Co., LTD. (Beijing, China). 1 × 10^6 HT22 cells/well cultured in 6-well plates, were transfected with 100pM siRNA according to the manufacturer’s method. After 24 h, the cells were treated with 20µM Idebenone for 8 h, and then collected for further analysis.

### Western Blot Analysis

1 × 10^6 HT22 cells/well cultured in 6-well plates were collected and lysed by adding a mixture of Radio Immunoprecipitation Assay (RIPA) lysis buffer (Dingguo Biotech Co., LTD, China), protease inhibitor and Phenyl methane sulfonyl fluoride (PMSF) (100:1:1) (Dingguo Biotech Co., LTD, China). The proteins were separated by 12% sodium dodecyl sulphate-polyacrylamide gel electrophoresis and transferred to a methanol-activated polyvinylidene fluoride membrane. The membranes were blocked in 5% skim milk for 2 h at 37℃ in a shaker, and then they washed with Tris Buffered Saline with Tween 20 (TBST) for 3 times. Rat anti-mouse P53 protein polyclonal antibody (Bioss Biotech Co., LTD, China), rabbit anti-mouse Caspase3 polyclonal antibody (Bioss Biotech Co., LTD, China), rabbit anti-mouse SIRT3 polyclonal antibody (Bioss Biotech Co., LTD, China), rabbit anti-mouse P53-AC monoclonal antibody (Abcam, England), and rabbit anti-mouse β-actin monoclonal antibody (Bioss Biotech Co., LTD, China) were separately incubated with protein-transferred membranes overnight at 4℃, and then they were washed with TBST for 3 times. Then horseradish peroxidase (HRP) secondary antibody was used to incubate for 2 h. Protein bands were developed with microporous enhanced chemiluminescence (ECL) Plus reagent and imaged on Tanon5500 image analysis system. The intensity of the strips was quantified using ImageJ software (U.S.A).

### RNA Extraction and Real-Time Quantitative PCR

1 × 10^6 HT22 cells were seeded in 6-well plates and RNA was extracted from these treated cells using TRIzol reagent (Dingguo Biotech Co., LTD, China). cDNA synthesis was performed using a FastKing one-step method to remove the first strand of genomic cDNA and synthesize a premix reagent (KR118) (Tiangen BioTech Co., LTD, China). The mRNA expression levels of SIRT3, P53, Caspase3 and β-actin were determined by PerfectStart® Green qPCR SuperMix (AQ601) (TransGen Biotech Co., LTD, China) and MX3000P real-time quantitative PCR system.

SIRT3 real-time quantitative polymerase chain reaction in mice primer (Sangon Biotech Co., LTD, China): Forward,5’-GCCCAATGTCACTCACTACTTCCTG-3’; Reverse,5’- CCACCAGCCTTTCCACACCATG-3’. Mouse P53 real-time quantitative polymerase chain reaction primer: Forward, 5’-TGAACCGCCGACCTATCCTTACC-3’; Reverse, 5’-CTAGGCTGGAGGCTGGAGTGAG-3’. Mice Caspase3 real-time quantitative polymerase chain reaction primer: Forward,5’-CACTGGAATGTCATCTCGCTCTGG–3’; Reverse, 5’-GTCGCCTCTGAAGAAGCTAGTCAAC-3’. Mouse β -actin real-time quantitative polymerase chain reaction primer: Forward, 5’-CGTGGCTACAGCTTCACCAC-3’; Reverse, 5’-TGGCCATCTCCTGCTCGAAG-3’. β-actin as normalized control. Real-time PCR amplification temperature was 95 °C, incubation time was 30s, and amplification cycle was 40 cycles (95 °C, 15s; 55 °C, 15s; 72 °C, 45s).

### NAD ^+^ / NADH Detection

HT22 cells (1 × 10^6 / sample) cultured in 6-well plates, were respectively treated with H_2_O_2_ (0 and 250µM); Idebenone (20µM), and a combination of these two drugs for 8 h. Cells were then collected using a NAD^+^/NADH extraction buffer solution. Intracellular NAD^+^ content was determined with a NAD^+^/NADH assay kit with WST-8 (2-(2-Methoxy-4-nitrophenyl)-3-(4-nitrophenyl)-5-(2,4-disulfophenyl)-2 H-tetrazolium Sodium Salt) (Beyotime Biotech Co., LTD, China), according to the manufacturer’s protocol. The NAD^+^/NADH ratio was calculated by using standard curves for NAD^+^ and NADH.

### Statistical Analysis

SPSS25.0 software (U.S.A) was used for data statistical analysis, including western blot, real-time quantitative PCR, cell viability assay, and NAD^+^/NADH detection. *P* < 0.05 obtained from the corresponding results was considered as statistically significant.

## Results

### Idebenone Protects against H_2_O_2_-Induced Neurotoxicity in HT22 Cells

**Fig. 1:**
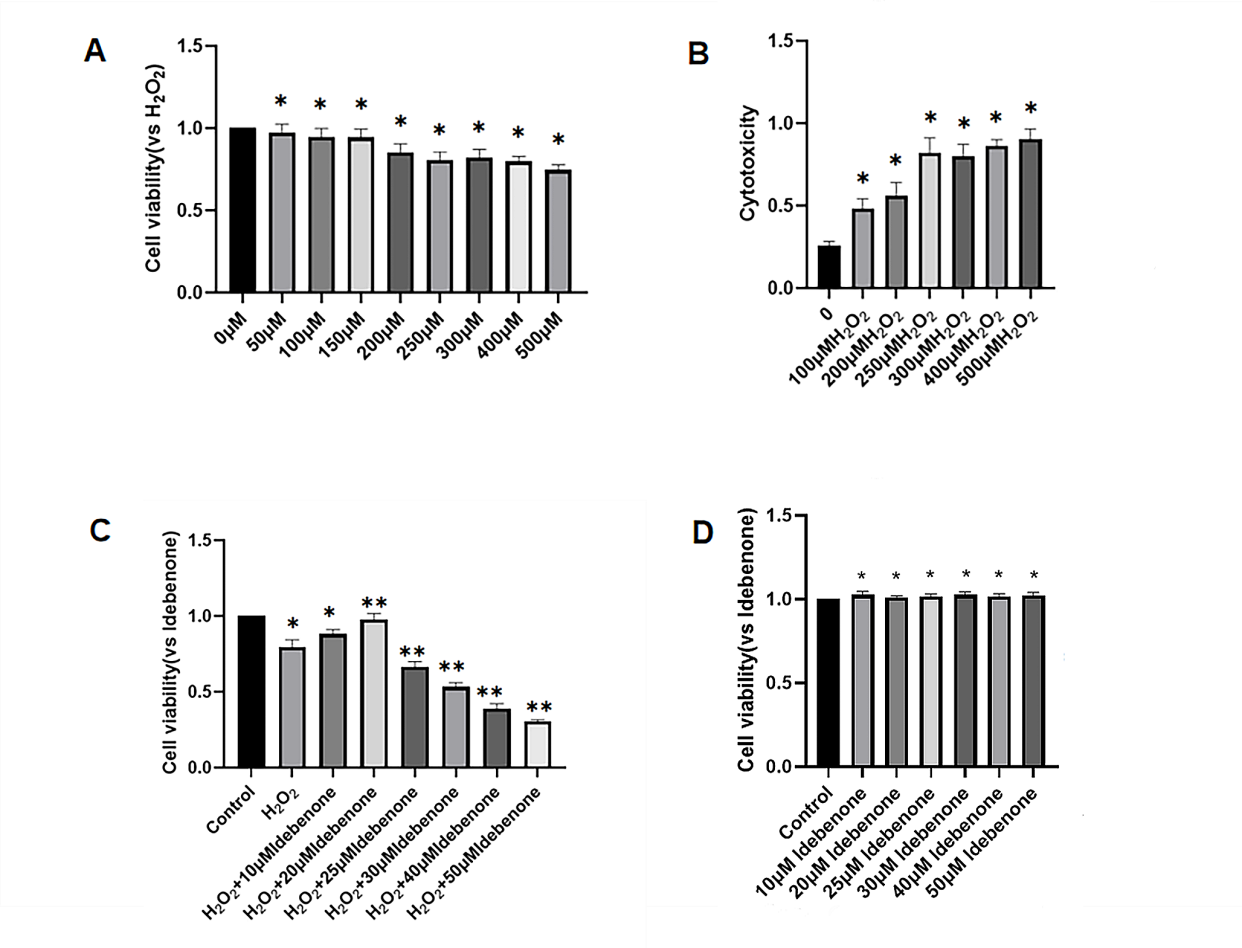
Idebenone Protects against H_2_O_2_-Induced Neurotoxicity in HT22 Cells. (**A**) MTT method was used to measure the survival rate of cells treated with different concentrations of H_2_O_2_. **p < 0.01, Compared with Control group. (**B**) Cytotoxicity of different concentrations of H_2_O_2_ as measured by LDH release assay, * *p* < 0.05, Compared with Control group. (**C**) MTT method was used to measure the survival rate of cells treated with different concentrations of Idebenone. * *p* < 0.05 and**p < 0.01,Compared with Control group. (**D**) MTT method was used to measure the survival rate of cells treated with different concentrations of Idebenone. *p < 0.05, Compared with Control group

HT22 cells were treated with a concentration gradient of H_2_O_2_ to determine the optimal concentration to construct the oxidative stress injury model in vivo. The results of MTT assays showed that there were no significant differences of the survival rate between 50, 100 and 150µM H_2_O_2_ treated group and control group (Fig. 1A). Cell viability decreased gradually beginning at 200µM and it reached to 70% at 250µM (Fig. 1A). Also, this effect was determined for the groups exposed to the same (unusual expression) 300µM and 400µM of H_2_O_2_. On the other hand, the cytotoxicity induced by H_2_O_2_ was measured by LDH release rate, results showed that the cytotoxicity increased significantly when 250µM and 300µM of H_2_O_2_ were assayed compared with that registered for 200 μm, reaching about 75% (Fig. 1B). Higher concentrations of 500µM of H_2_O_2_ significantly decreased cell viability, nearly 60%. Therefore, 250µM H_2_O_2_ was chosen to perform the oxidative stress injury model in HT22 cells.

Then, the HT22 cells were pretreated with different dosages of Idebenone, the results showed that HT22 cells treated with 10 and 20µM Idebenone had higher survival rate than those treated with H_2_O_2_ alone. It reached to 95% in the 20µM Idebenone group (Fig. 1C). Notably, when the concentration of Idebenone exceeds 25µM, the survival rate of cells decreased with the increase of Idebenone concentration. It has been confirmed that the toxicity of Idebenone gradually appears in a dose-dependent manner after the concentration of Idebenone exceeds 25µM. Additionally, the effect of Idebenone on the survival rate of normal cells has also been explored (Fig. 1D). The results indicate that, in the absence of H_2_O_2_ damage, different concentrations of Idebenone do not have a significant impact on cell survival.

### Idebenone Reduces Apoptosis of HT22 Cells after Oxidative Stress Injury

**Fig. 2:**
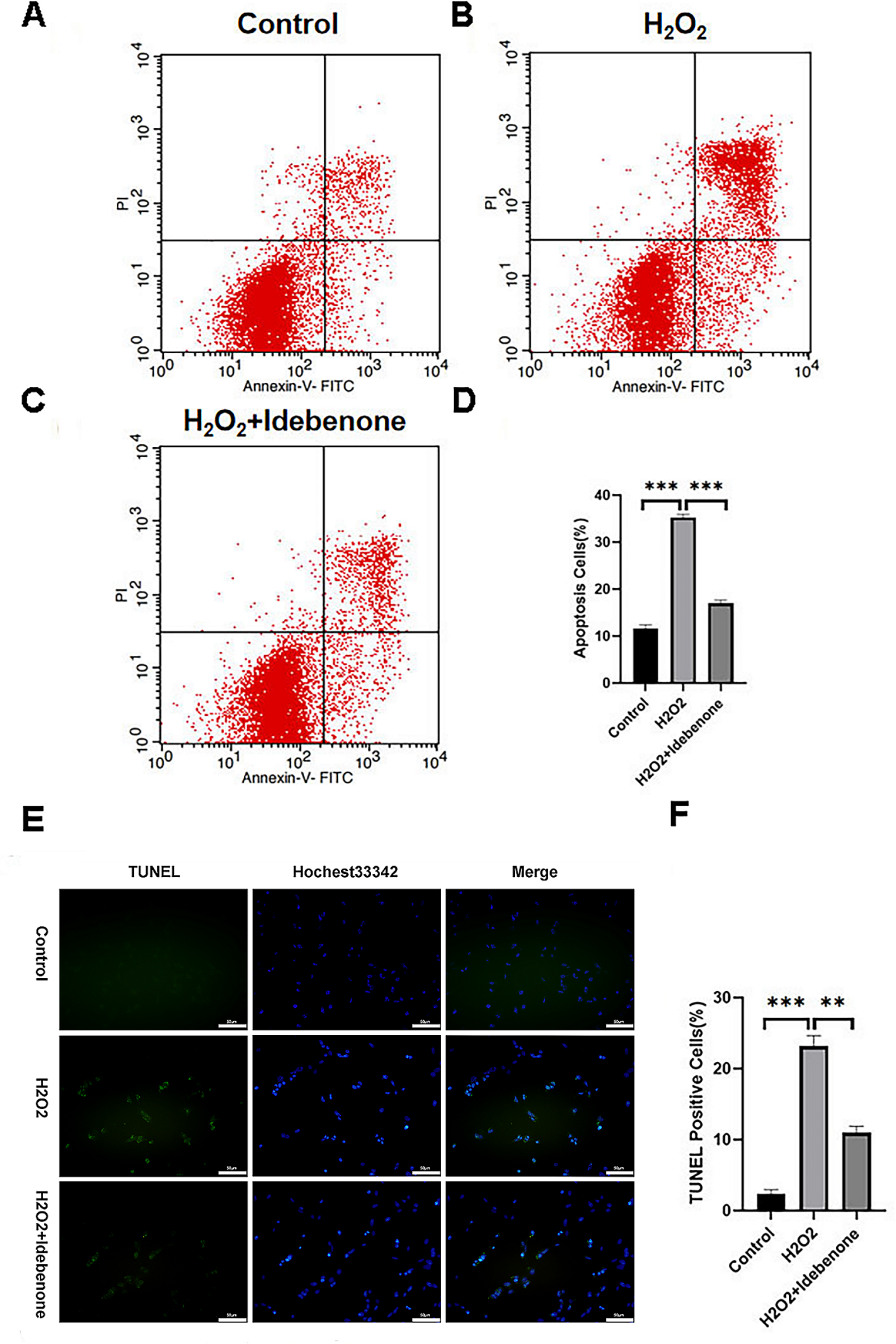
Idebenone reduces apoptosis of HT22 cells after oxidative stress injury. (**A**)(**B**)(**C**) Flow cytometry test result. (**D**) Statistical analysis of cell apoptosis rate. (**E**) The morphology of HT22 cells in each group was observed under different fluorescence microscope. Scale bar: 50 μm. (**F**) Statistical results of TUNEL positive cell rate, *p < 0.05, **p < 0.01 and ***p < 0.001

In order to verify the protective effect of Idebenone on oxidative stress-induced apoptosis of HT22 cells, HT22 cells were divided into control group, H_2_O_2_ injury group and Idebenone pretreatment group. Flow cytometry and TUNEL staining were used to detect the apoptosis of HT22 cells. Flow cytometry results showed that the apoptosis rate of HT22 cells in the H_2_O_2_ injury group was significantly increased compared with the control group (Fig. 2A, B, C and D). Compared with H_2_O_2_ injury group, the apoptosis rate of HT22 cells in Idebenone pretreatment group was significantly decreased (*P* < 0.001). The TUNEL staining results showed that compared with the control group, the TUNEL positive cell rate in the H_2_O_2_ injured group was significantly increased (*P* < 0.001) (Fig. 2E and F). Compared with H_2_O_2_ injury group, TUNEL positive cell rate in Idebenone pretreatment group decreased, but was still higher than that in control group (*P* < 0.01).

### Idebenone Plays a Protective Role by Reducing the Expression of Apoptotic Protein P53/Caspase3

**Fig. 3:**
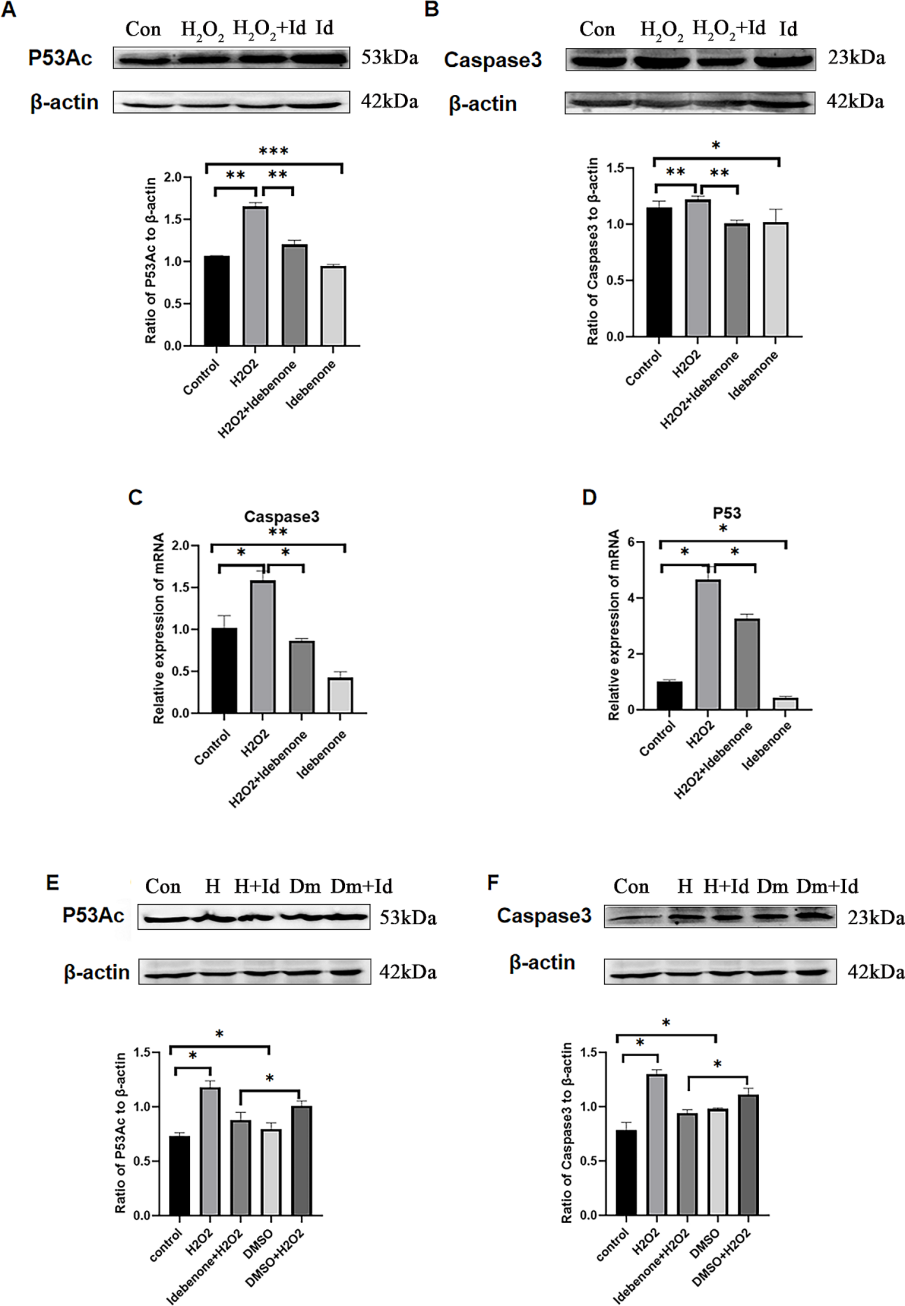
Idebenone Plays a Protective Role by Reducing the Expression of Apoptotic Protein P53/Caspase3. (**A**)(**E**) The expression of P53Ac protein was detected by western blot. (**B**)(**F**) The expression of Caspase3 protein was detected by western blot. (**C**) The expression of Caspase3 protein was detected by real-time PCR. (**D**) The expression of P53 protein was detected by real-time PCR,*p < 0.05, **p < 0.01 and ***p < 0.001

To investigate whether P53 is involved in the protective effect of Idebenone, the expression level of apoptosis-related proteins P53 and Caspase3 was detected in different groups, including control group (Con), H_2_O_2_ treated group(H_2_O_2_), Idebenone treated group (Id) and H_2_O_2_ and Idebenone both treated group(H_2_O_2_ + Id). Western blot and real-time quantitative PCR results showed that the expression level of apoptosis related protein P53 and its activated form P53Ac (Acetylated P53) were up-regulated in the H_2_O_2_ group compared with the control group, while the addition of Idebenone pretreatment could down-regulate the expression level of P53 and P53Ac in HT22 cells after H_2_O_2_ injury (Fig. 3A and D). Among them, the expression level of P53 and P53Ac in the group Id was also significantly lower than that in the control group. In addition, the expression level of Caspase3 protein in each experimental group was consistent with the trend of P53 and P53Ac, and the expression level of Caspase3 protein was up-regulated after H_2_O_2_ injury while being down-regulated after Idebenone pretreatment (Fig. 3B and C). It is worth noting that when DMSO was added to cells alone, the expression of P53 protein and Caspase3 protein was not found to decrease compared with the control group (Fig. 3E and F). However, the expression of P53 and Caspase3 proteins was decreased when Idebenone was added to H_2_O_2_ damaged cells with DMSO solution (Fig. 3E and F). This also indicated that the addition of DMSO had no significant effect on the therapeutic effect of Idebenone.

### Prediction of Potential Targets of Idebenone by Network Pharmacology

**Fig. 4:**
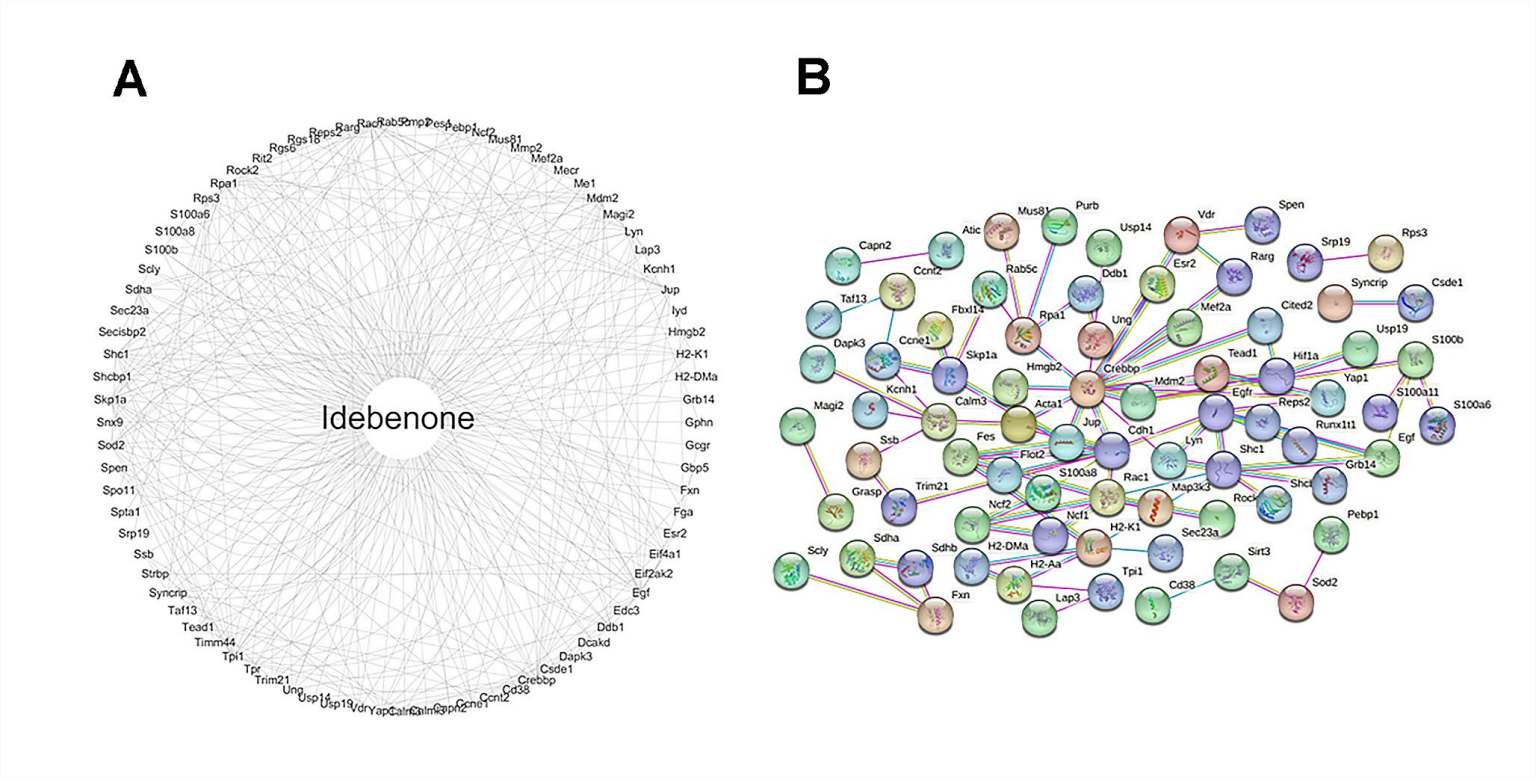
Prediction of potential targets of Idebenone by network pharmacology. (**A**)Cytoscape to visualize potential targets of Idebenone. (**B**) Potential targets of Idebenone and PPI networks of SIRT3

In order to explore which proteins are involved in the process of playing the role of anti-oxidative stress, potential targets of Idebenone were analyzed with network pharmacology, and 270 potential targets were screened using the 3D structure of Idebenone (Fig. 4A). Since the mitochondrial deacetylase SIRT3 has been demonstrated to deacetylate and inactivate the activated P53 protein, it was hypothesized to be associated with the down-regulation of P53Ac expression by Idebenone in the anti-oxidative stress process. Therefore, PPI protein interaction network was used in this study to investigate the relationship between these 270 genes and SIRT3. The results showed that the membrane protein CD38 and superoxide dismutase SOD2 had direct effects on SIRT3 among the 270 potential targets (Fig. 4B). While, SOD2 is often studied as a downstream protein of SIRT3. It has been reported that Idebenone can scavenge accumulated ROS and reduce cell death by up-regulating the expression of SIRT3 and SOD2. However, the mechanism of whether Idebenone affects the expression level of SIRT3 through CD38 is not clear, so CD38 was chosen for subsequent studies.

**Fig. 5:**
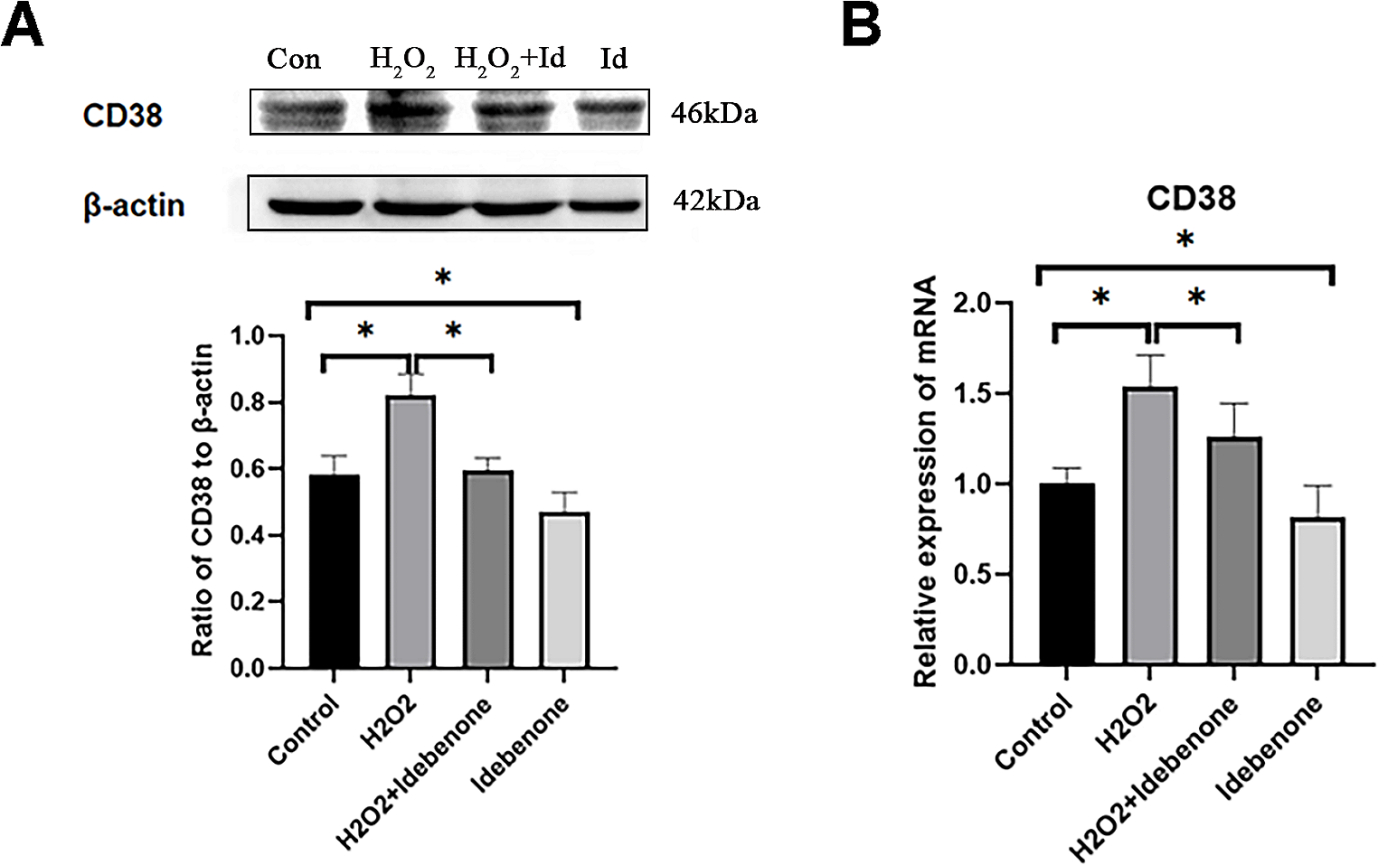
Idebenone Down-regulates CD38 Expression in H_2_O_2_-damaged HT22. (**A**) The expression of CD38 protein was detected by western blot. (**B**) The expression of CD38 protein was detected by real-time PCR. *p < 0.05, **p < 0.01 and ***p < 0.001

**Idebenone Down-regulates CD38 Expression in H**_**2**_**O**_**2**_**-damaged HT22** Western blot and real-time quantitative PCR were used to detect the expression of CD38, which was divided into the control group, H_2_O_2_ damaged group, Idebenone treated group and H_2_O_2_ and Idebenone both treated group (Fig. 5A and B). The results showed that CD38 expression in H_2_O_2_ damaged group was significantly higher than in the control group, while the CD38 expression in H_2_O_2_ and Idebenone both treated group was significantly lower than in the H_2_O_2_ damaged group (Fig. 5A). In addition, the expression of CD38 protein in Idebenone treated group was also significantly down-regulated.

**Fig. 6:**
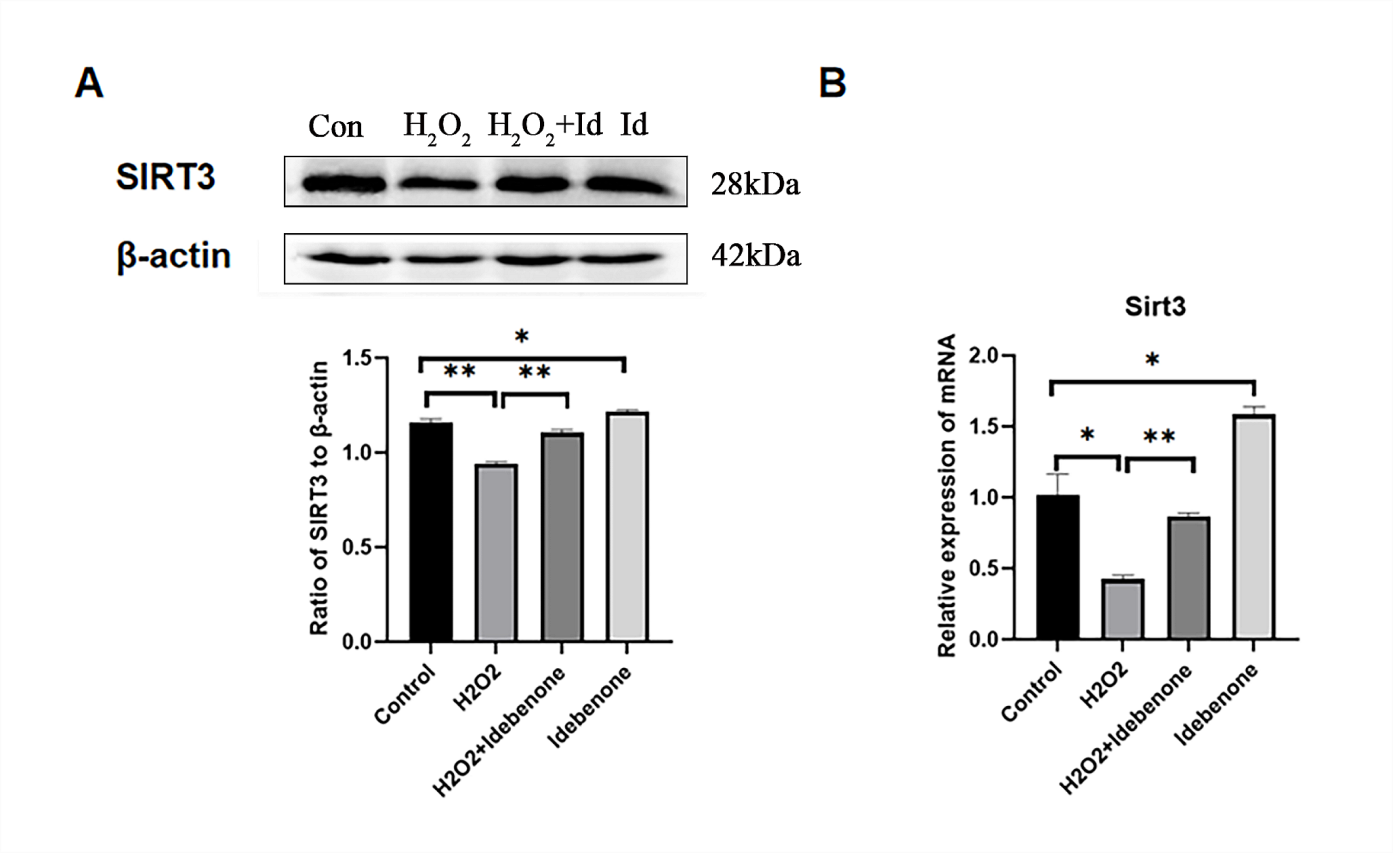
Idebenone Upregulates SIRT3 Expression in H_2_O_2_-damaged HT22 Cells. (**A**) The expression of SIRT3 protein was detected by western blot. (**B**) The expression of SIRT3 protein was detected by real-time PCR. *p < 0.05, **p < 0.01 and ***p < 0.001

**Idebenone Upregulates SIRT3 Expression in H**_**2**_**O**_**2**_**-damaged HT22 Cells** Western blot and real-time quantitative PCR were used to detect the expression of SIRT3 in four groups including the control group, the H_2_O_2_ damaged group, the Idebenone treated group and the H_2_O_2_ and Idebenone both treated group(Fig. 6A and B). The results showed that the SIRT3 expression in the H_2_O_2_ damaged group was significantly lower than in the control group, while the SIRT3 expression in the H_2_O_2_ and Idebenone both treated group was significantly higher than in the H_2_O_2_ damaged group (Fig. 6A). In addition, the expression of SIRT3 protein in Idebenone treated group was also significantly up-regulated(Fig. 6B).

### Idebenone Can Help HT22 Cells Release NAD^+^

**Fig. 7:**
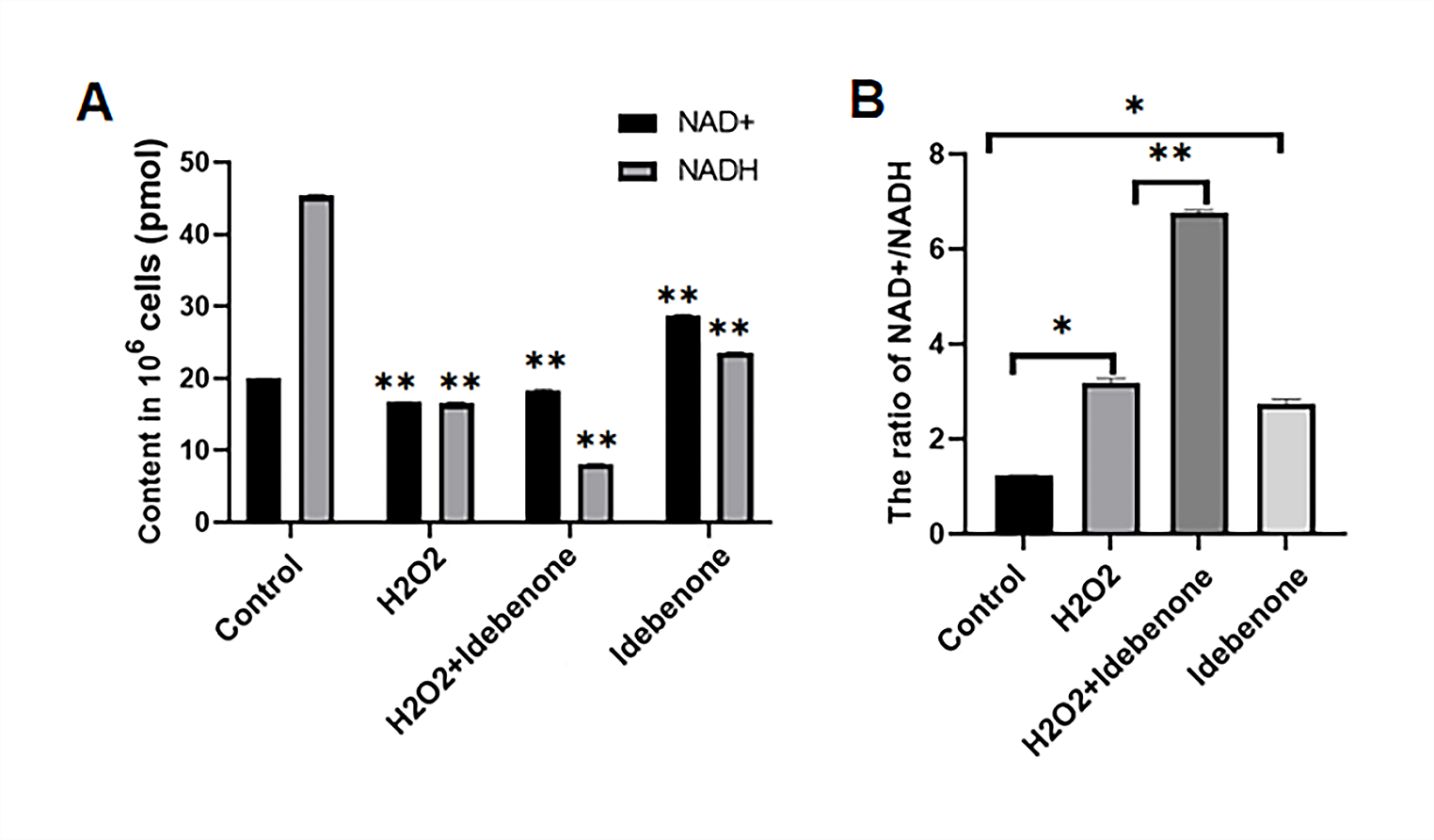
Idebenone Can Help HT22 Cells Release NAD^+^. (**A**) NAD^+^ and NADH contents were measured by NAD^+^/NADH kit. (**B**) NAD^+^/NADH ratio of cells was measured by NAD^+^/NADH kit. *p < 0.05, **p < 0.01

To further verify the relationship between the down-regulation of CD38 and the up-regulation of SIRT3, NAD^+^/NADH detection kit of WST-8 was used to determine the level of NAD^+^ in cells of the control group, the H_2_O_2_ damaged group, the Idebenone treated group and the H_2_O_2_ and Idebenone both treated group. The results showed that the concentration of NADH was significantly higher than that of NAD^+^ in the control group, and the concentration of NAD^+^ and NADH was almost the same in the H_2_O_2_ injured group (Fig. 7A and B). The ratio of NAD^+^ to NADH in the H_2_O_2_ injured group was higher than that in the control group, while the concentration of NAD^+^ in the Idebenone pretreated group was significantly higher than that of NADH. Therefore, the NAD^+^/NADH ratio increased more significantly than that of the H_2_O_2_ injury group (Fig. 7A). In addition, compared with the control group, the concentration of NAD^+^ was higher than that of NADH, and the NAD^+^/NADH ratio was also higher in the group Idebenone added alone (Fig. 7B).

### Idebenone Induces the Expression of SIRT3 in SIRT3-Down-Regulated HT22 Cells and the Deacetylation of P53Ac

**Fig. 8:**
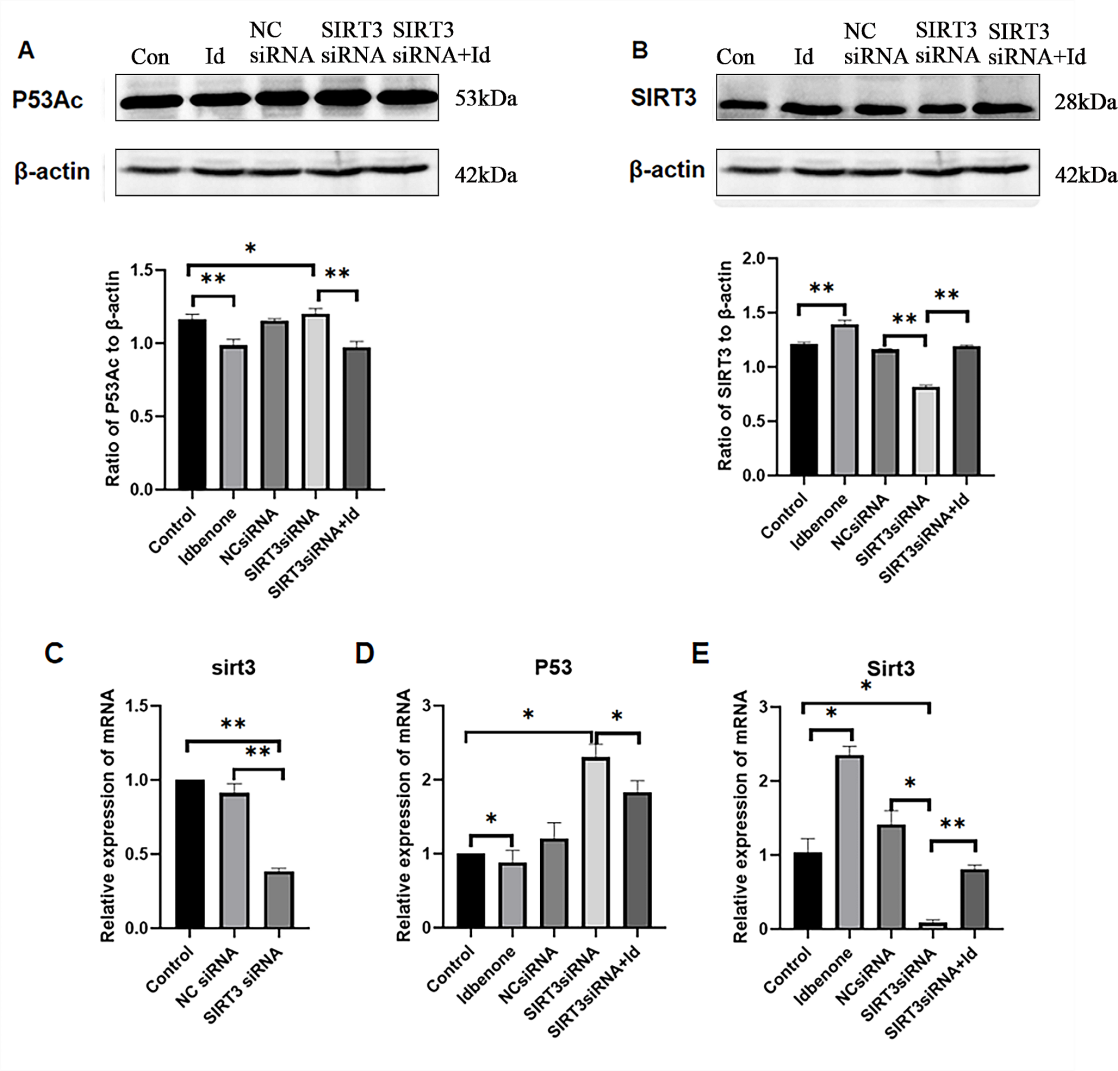
Idebenone Induces the Expression of SIRT3 in SIRT3-Down-Regulated HT22 Cells and the Deacetylation of P53Ac. (**A**) The expression of P53Ac protein was detected by western blot after SIRT3 down-regulated in various groups. *p < 0.05, **p < 0.01 and ***p < 0.001 (**B**) The expression of SIRT3 protein was detected by western blot after SIRT3 down-regulated in various groups. *p < 0.05, **p < 0.01 and ***p < 0.001 (**C**) The expression of SIRT3 was detected by real-time PCR after SIRT3 down-regulated. *p < 0.05, **p < 0.01 and ***p < 0.001 (**D**) The expression of P53 protein was detected by real-time PCR after SIRT3 down-regulated in various groups. *p < 0.05, **p < 0.01 and ***p < 0.001 (**E**) The expression of SIRT3 protein was detected by real-time PCR after SIRT3 down-regulated in various groups. *p < 0.05, **p < 0.01 and ***p < 0.001

In order to further explore whether SIRT3 is directly related to P53Ac, and the effect of Idebenone on SIRT3-knockout HT22 cells, HT22 cells were transfected with SIRT3 siRNA to down-regulate the expression of SIRT3 protein. And the negative control was transected with NC siRNA. The results showed that the SIRT3 protein expressed in HT22 cells transfected with SIRT3 siRNA3 was significantly lower than that in the NC siRNA group, demonstrating the effectiveness of SIRT3 siRNA (Fig. 8C). Afterwards, HT22 cells and SIRT3 siRNA transfected HT22 cells were treated with Idebenone respectively. The expression level of SIRT3 and P53 protein was detected by western blot and real-time quantitative PCR. The results showed that the expression level of SIRT3 protein decreased more obviously in the SIRT3 siRNA group compared with the control group (Fig. 8B and E). Meanwhile, there was an upward trend in P53 protein expression (Fig. 8A and D). Compared with the SIRT3 siRNA group, SIRT3 was significantly up-regulated and P53 was significantly down-regulated in the SIRT3 siRNA group pretreated with Idebenone. In addition, compared with the control group, the expression level of SIRT3 protein was up-regulated and the expression level of P53 protein showed the opposite direction in the group Idebenone added alone.

## Discussion

Oxidative stress injury refers to a pathological condition, which can increase the production of reactive oxygen species (ROS) in the body, leading to mitochondrial dysfunction [1], causing neuron damage, increasing neuron deaths, and is associated with a variety of neuron injury diseases, such as Parkinson’s disease and Alzheimer’s disease [4]. Hydrogen peroxide(H_2_O_2_) is a common reagent for inducing oxidative stress injury assays [23]. HT22 hippocampal nerve cell line has been proved to be an ideal option for studying nerve oxidative stress injury diseases [24]. In this study, H_2_O_2_ was applied to HT22 cells to stablish the in-vitro nerve oxidative stress injury models. It was found that H_2_O_2_ dramatically inhibited the proliferation of HT22 cells. Moreover, H_2_O_2_ induced a large amount of apoptosis in HT22 cells. Combining with the MTT and microscopic results, 250µM H_2_O_2_ was chosen to construct the neuronal oxidative stress injury model of HT22 cells.

Idebenone, a coenzyme Q analogue, can participate in the electron transport chain by enhancing NADH dehydrogenase activity [25]. Studies have shown that it can reduce the neuronal damage and have a protective effect on oxidative stress neurons, which has been applied in clinical practice [26]. However, the mechanism of how Idebenone fights oxidative stress and reduces neuronal death remains unclear. In this study, 250µM H_2_O_2_ cell injury model was pretreated with different concentrations of Idebenone. The results showed that Idebenone could dose-dependently enhance HT22 cells viability after H_2_O_2_ induced injury, which indicated that it could reduce cytotoxicity to protect cells from ROS injury. However, Idebenone showed cytotoxicity in a dose-dependent manner [27]when exceeded 25µM. Therefore, 20µM was determined as the optimal concentration of Idebenone for observation.

Subsequently, flow cytometry and TUNEL staining was used to detect the apoptosis of HT22 cells before and after Idebenone was added to the H_2_O_2_ damage. It was confirmed that oxidative stress damage can cause neuronal cell apoptosis, and Idebenone can counteract oxidative stress damage by reducing cell apoptosis. In addition, this study also detected the expression of apoptosis-related proteins P53 and Caspase3 in the Idebenone pretreated HT22 cell oxidative stress model.

The P53 gene is a human tumor suppressor gene [28]. The activation of P53 induces cell apoptosis, and its activity is regulated by various post-translational modifications such as phosphorylation, acetylation, ubiquitination, and methylation [14, 15,16]. Studies have shown that P53 acetylation can accelerate cell apoptosis, and inhibit tumor growth, that is, P53Ac is the active form of the P53 protein. The Caspase family is a key component in the process of cell apoptosis, and its activation and overexpression both cause cell apoptosis. Caspase3 is the most common one and is often used as a marker protein to detect cell apoptosis [29]. In addition, a large number of studies have shown that Caspase3 is one of the downstream molecules of P53. The RT-qPCR results showed that the mRNA expression levels of apoptosis-related proteins P53 and Caspase3 in the H_2_O_2_ group were upregulated, and the mRNA expression levels of apoptosis-related proteins P53 and Caspase3 in the Idebenone pretreated group were significantly decreased, further proving that Idebenone can counteract oxidative stress damage by reducing cell apoptosis. It is worth noting that the Western Blot results showed that Idebenone can reduce the increase in P53Ac protein expression caused by H_2_O_2_, indicating that Idebenone reduces cell apoptosis and is related to the deacetylation activation of P53 protein. Therefore, Idebenone exerts a protective effect on neuronal cells by deacetylating P53 in mitochondria. However, which deacetylase is involved in this process still needs further exploration to verify.

SIRT3 is a member of the deacetylase protein family and is a NAD^+^-dependent deacetylase that primarily exists in mitochondria [10]. Studies have shown that the application of idebenone upregulates the expression of SIRT3, which protects vascular endothelial cells from lipid peroxidation through the SIRT3-SOD2-mtROS pathway[8]. Moreover, SIRT3, along with its family members SIRT1 and SIRT6, can reduce the expression of the apoptosis protein P53 [17, 18, 30]. The activation of mitochondrial deacetylase SIRT3 was suspected to involve in the deacetylation of P53 protein that occurs during the application of Idebenone to combat oxidative stress[9]. In this study, network pharmacology was used to predict the potential gene targets of Idebenone. The screened genes were studied for protein-protein interactions (PPI) with SIRT3. The results showed that the membrane protein CD38 and the superoxide dismutase SOD2 can interact with SIRT3. Since it is clear that SIRT3 directly clears ROS by regulating SOD2 activity and reducing cell death, and SOD2 is not related to the deacetylation of P53, it is not the focus of this study[11–13].

CD38 is a transmembrane glycoprotein that is widely expressed on the surface of immune cells and neuronal cells, and is closely related to oxidative stress [7]. In addition, the expression level of CD38 is closely related to the concentration of NAD^+^. An increase in CD38 can promotes CD38-dependent NAD hydrolysis enzyme activity, leading to a decrease in NAD^+^ concentration, while inhibition of CD38 can increase NAD^+^ concentration [31]. Studies have shown that by inhibiting the expression of CD38 and increasing NAD^+^ concentration, SIRT3 concentration can be increased to clear ROS and reduce lipid accumulation, thereby inhibiting the development of non-alcoholic fatty liver disease [32]. This suggests that CD38 and SIRT3 may interact with each other through NAD^+^[5, 6]. In this study, the expression of CD38 in HT22 cells was detected after oxidative stress damage and Idebenone intervention to verify the effect of Idebenone on CD38. The results showed that oxidative stress damage increased CD38 expression, while the application of Idebenone reduced CD38 expression after oxidative stress damage, confirming the previous prediction that CD38 is one of the targets of Idebenone.

In addition, the concentration of NAD^+^ and NAD^+^/NADH values was detected in HT22 cells after Idebenone inhibited oxidative stress damage. The results showed that the NAD^+^/NADH value in the idebenone pretreatment group was significantly higher than that in the H_2_O_2_ damage group, indicating that Idebenone accelerated the conversion of NADH to NAD^+^, improving the efficiency of the electron transport chain. This acceleration effect was mainly due to the inhibition of CD38 expression and the suppression of NAD^+^ decomposition.

The expression of SIRT3 was also detected in HT22 cells before and after oxidative stress damage and Idebenone treatment. The results showed that the expression of SIRT3 was severely decreased in the oxidative stress-injured HT22 cells, while application of Idebenone restored SIRT3 expression, which was consistent with the previous prediction. It suggests that Idebenone may upregulate SIRT3 by inhibiting CD38. This was further confirmed by the decrease in CD38 expression and the increase in SIRT3 expression in the Idebenone group.

To further verify the relationship between P53 and SIRT3, siRNA was conducted to down-regulate the SIRT3 expression in HT22 cells, and the expression of P53Ac and SIRT3 was detected before and after Idebenone treated. After SIRT3 down-regulated, the expression of P53 in HT22 cells showed an upward trend, indicating that SIRT3 directly participated in the activation of P53 deacetylation. Therefore, Idebenone inhibits CD38, increases NAD^+^ concentration, induces the up-regulation of SIRT3, and deacetylates P53Ac, finally reducing oxidative stress-induced HT22 cell apoptosis.

These results suggest that Idebenone counteracts oxidative stress-induced apoptosis in neuronal cells through the CD38-SIRT3-P53 pathway. Idebenone plays a positive role in inhibiting CD38 expression. It provides a new idea for clinical anti-oxidative stress injury diseases. Also it gives a informative value for the treatment of some CD38 immune-related diseases, such as lymphoma and multiple myeloma. However, this study has only established the connection between Idebenone and CD38, SIRT3, and P53, and more research is needed to explore the genes and signaling factors involved in this signaling pathway.

## Conclusion

This finding suggests that Idebenone plays an active role in promoting SIRT3 expression, which may provide new insights into the function of Idebenone and the role of SIRT3. It is informative for the use of Idebenone in the treatment of some SIRT3-related diseases, such as brain injury and mitochondrial dysfunction. Nevertheless, this study preliminarily established a link between Idebenone and SIRT3 and P53. More researches are needed to investigate potential signaling pathways.

### Electronic Supplementary Material

Below is the link to the electronic supplementary material.

Supplementary Material 1

## Data Availability

No datasets were generated or analysed during the current study.
