# Supplementary material for: Idebenone Antagonizes P53-Mediated Neuronal Oxidative Stress Injury by Regulating CD38-SIRT3 Protein Level
Source: Neurochem Res. 2024 Jun 12;49(9):2491–504. doi: 10.1007/s11064-024-04189-7 (PMC11310240; doi:10.1007/s11064-024-04189-7)
Supplement: Supplementary file 1 — Supplementary Material 1 [file 11064_2024_4189_MOESM1_ESM.pdf]

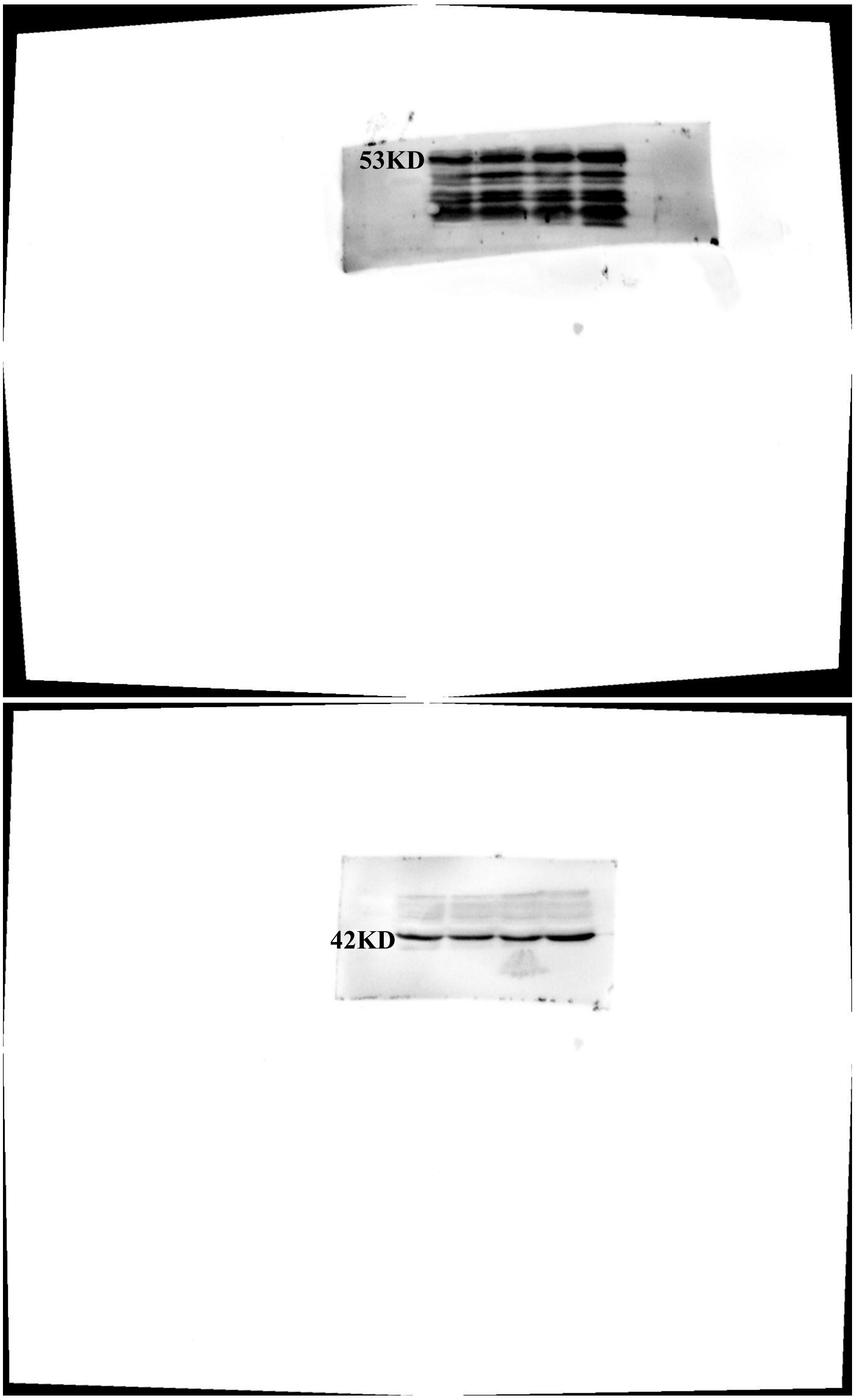

Figure3A. Western blot detected the expression of P53Ac and β-actin: They come from the same batch, the same sample, the same concentration, the same loading quantity, but not on the same membrane. Because the molecular weight of P53 and β-actin protein is too close to wash down.

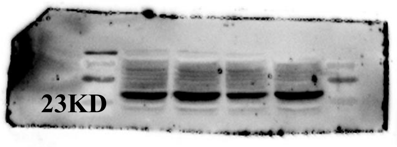

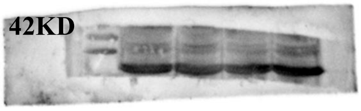

Figure3B. Western blot detected the expression of Caspase3 and β-actin :They come from the same batch, the same sample, the same concentration, the same loading quantity, the same membrane. We cut it out for simultaneous incubation.

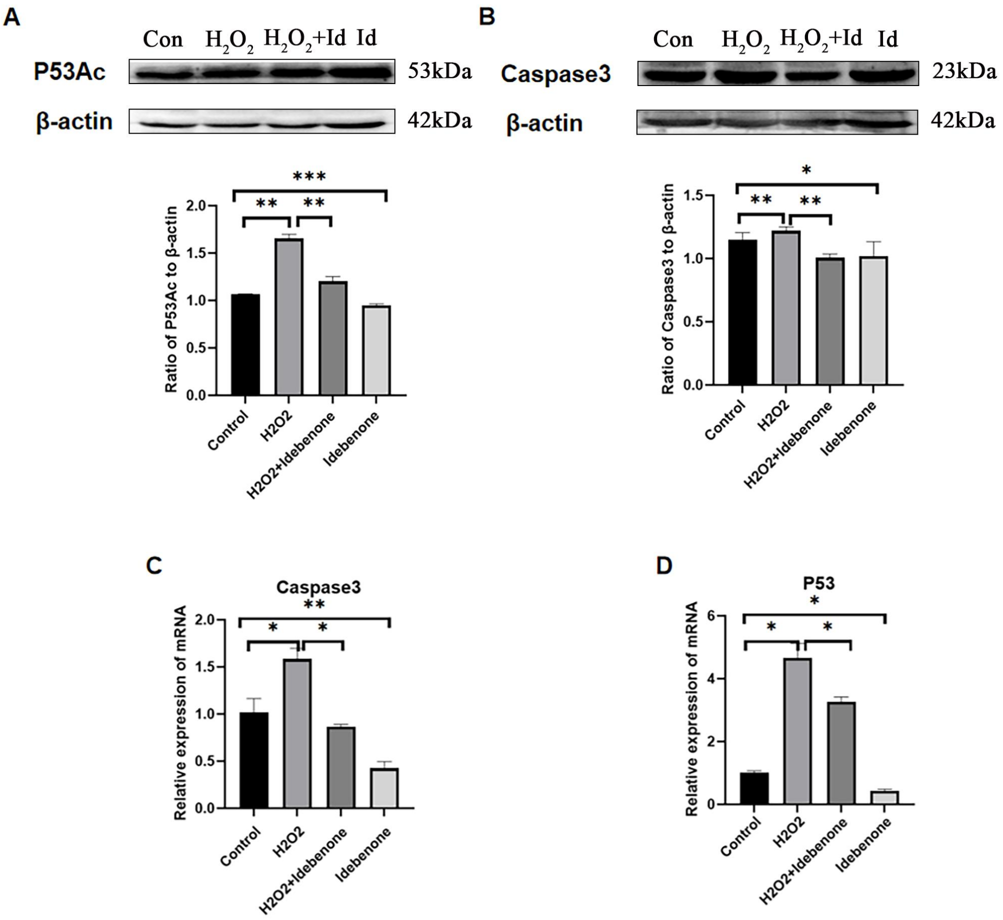
A
Con
H_2O_2
H_2O_2+Id
Id
P53Ac
53kDa
β-actin
42kDa
Ratio of P53Ac to β-actin
2.0
1.5
1.0
0.5
0.0
***
**
**
Control
H2O2
H2O2+Idebenone
Idebenone
B
Con
H_2O_2
H_2O_2+Id
Id
Caspase3
23kDa
β-actin
42kDa
Ratio of Caspase3 to β-actin
1.5
1.0
0.5
0.0
*
**
**
Control
H2O2
H2O2+Idebenone
Idebenone
C
Caspase3
Relative expression of mRNA
2.0
1.5
1.0
0.5
0.0
**
*
*
Control
H2O2
H2O2+Idebenone
Idebenone
D
P53
Relative expression of mRNA
6
4
2
0
*
*
*
Control
H2O2
H2O2+Idebenone
Idebenone

53KD
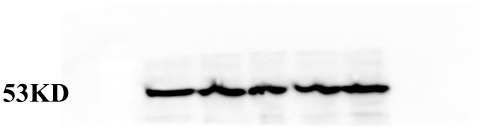

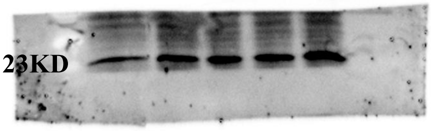

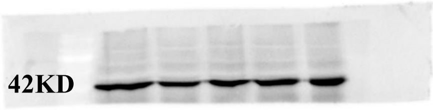

Figure3E、F. Western blot detected the expression of P53Ac and β-actin .Western blot detected the expression of Caspase3 and β-actin :They come from the same batch, the same sample, the same concentration, the same loading quantity, the same membrane. We cut it out for simultaneous incubation.

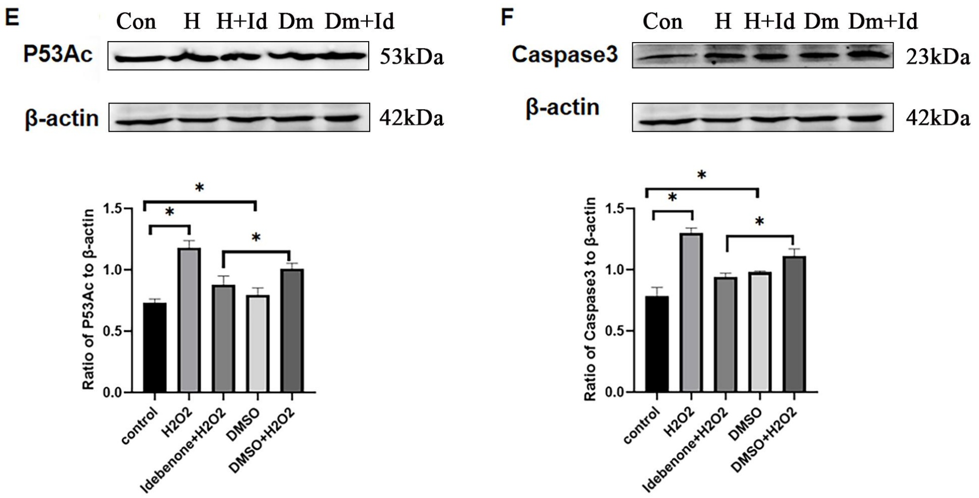
E
Con H H+Id Dm Dm+Id
P53Ac
53kDa
β-actin
42kDa
Ratio of P53Ac to β-actin
1.5
1.0
0.5
0.0
control
H2O2
Idebenone+H2O2
DMSO
DMSO+H2O2
F
Con H H+Id Dm Dm+Id
Caspase3
23kDa
β-actin
42kDa
Ratio of Caspase3 to β-actin
1.5
1.0
0.5
0.0
control
H2O2
Idebenone+H2O2
DMSO
DMSO+H2O2

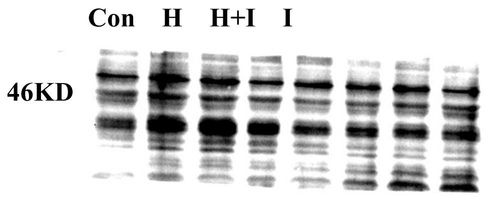
Con H H+I I
46KD

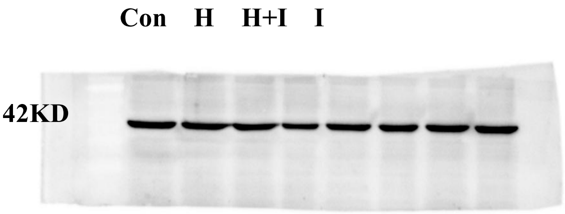

Figure5A. Western blot detected the expression of CD38 and β-actin .Western blot detected the expression of Caspase3 and β-actin :They come from the same batch, the same sample, the same concentration, the same loading quantity, the same membrane.

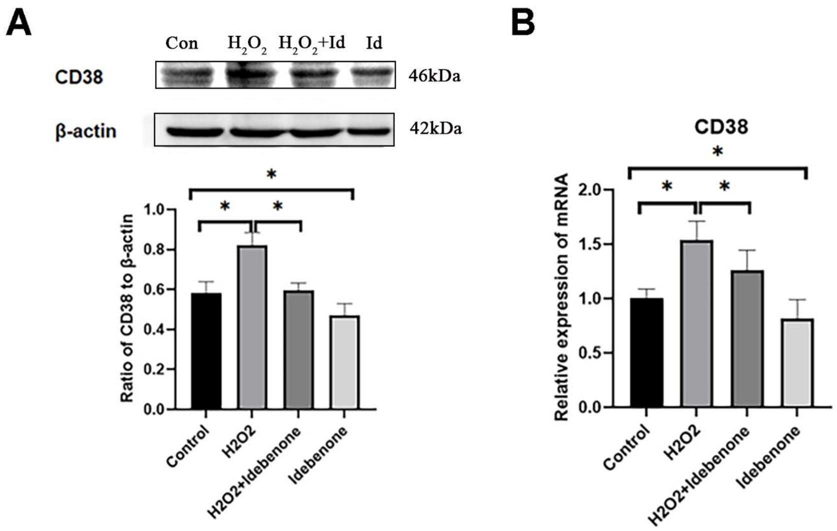

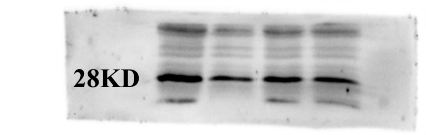

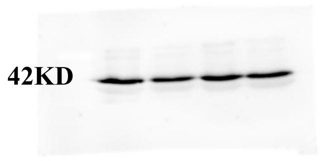

Figure6. Western blot detected the expression of SIRT3 and β-actin :They come from the same batch, the same sample, the same concentration, the same loading quantity, the same membrane.

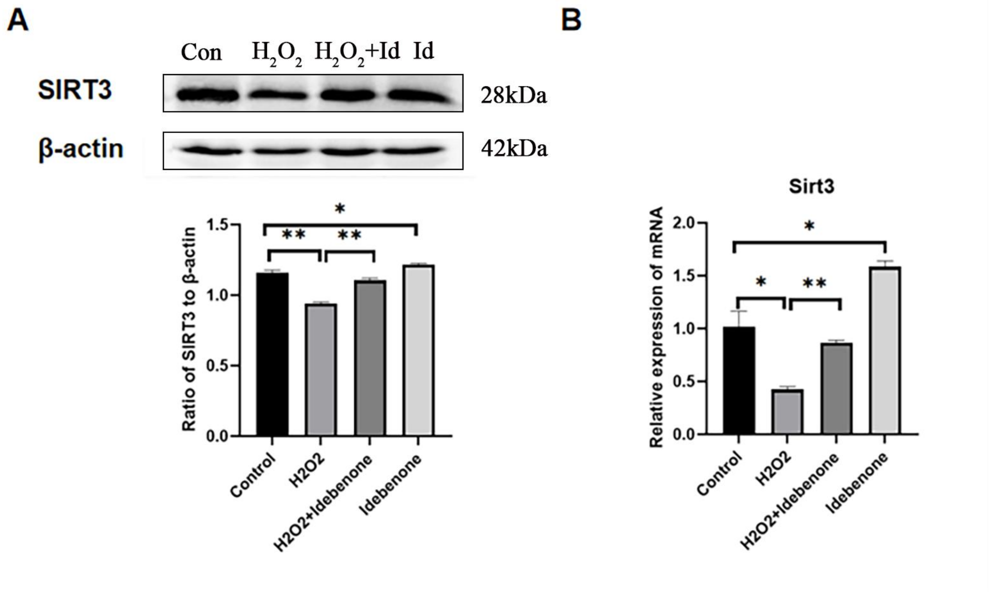

A
Con
H_2O_2
H_2O_2+Id
Id
SIRT3
28kDa
β-actin
42kDa
Ratio of SIRT3 to β-actin
1.5
1.0
0.5
0.0
*
**
**
Control
H2O2
H2O2+Idebenone
Idebenone
B
Sirt3
Relative expression of mRNA
2.0
1.5
1.0
0.5
0.0
*
*
**
Control
H2O2
H2O2+Idebenone
Idebenone

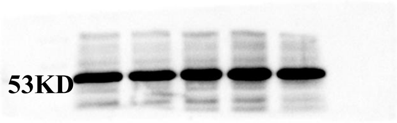

53KD

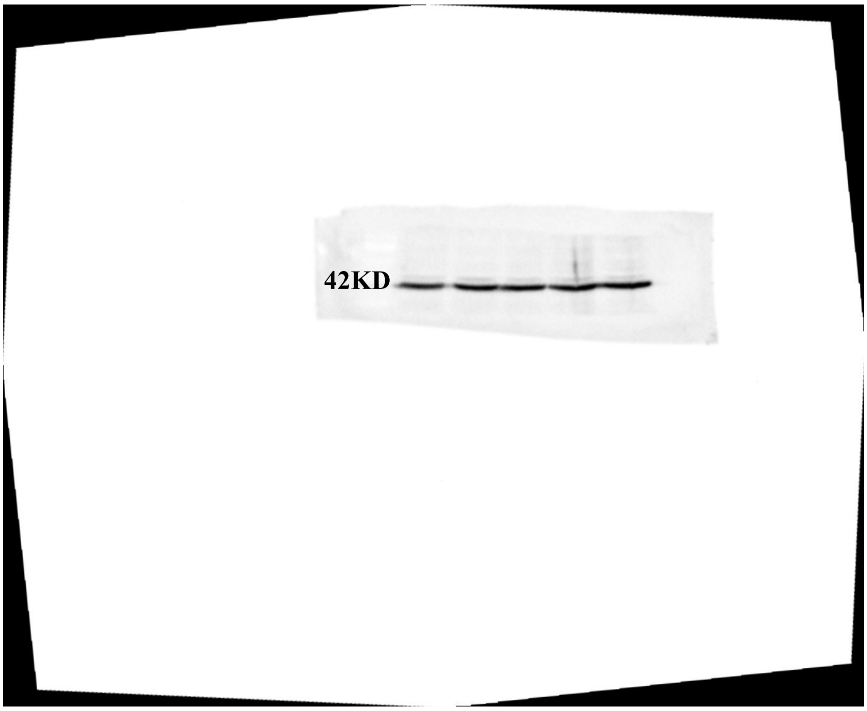

Figure8A. Western blot detected the expression of P53Ac and β-actin: They come from the same batch, the same sample, the same concentration, the same loading quantity, but not on the same membrane. Because the molecular weight of P53 and β-actin protein is too close to wash down.

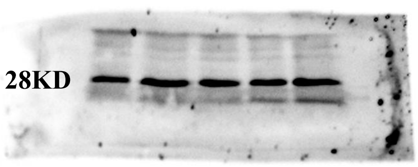

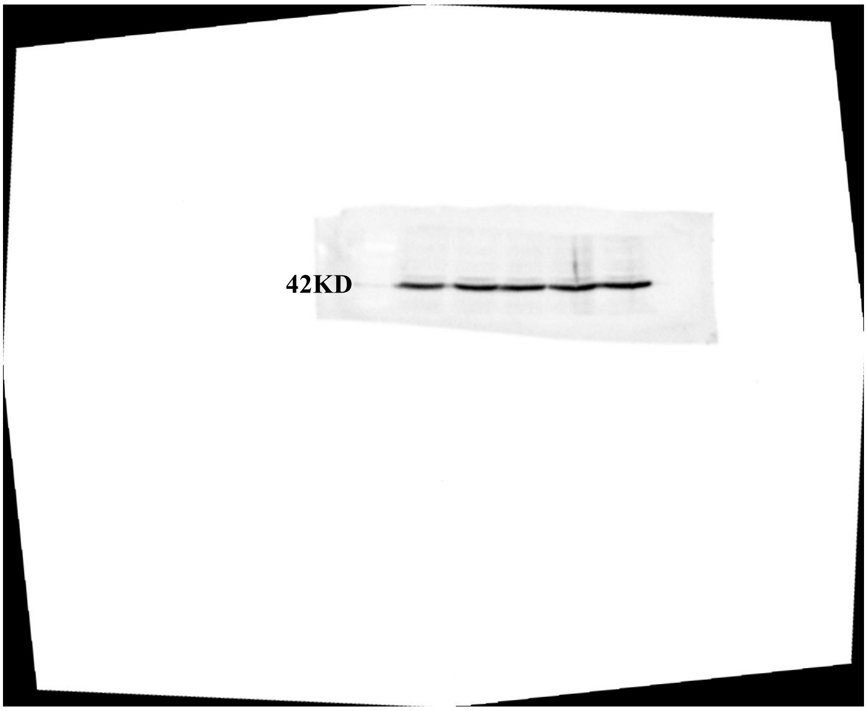

Figure8B.Western blot detected the expression of SIRT3 and β-actin :They come from the same batch, the same sample, the same concentration, the same loading quantity, the same membrane.

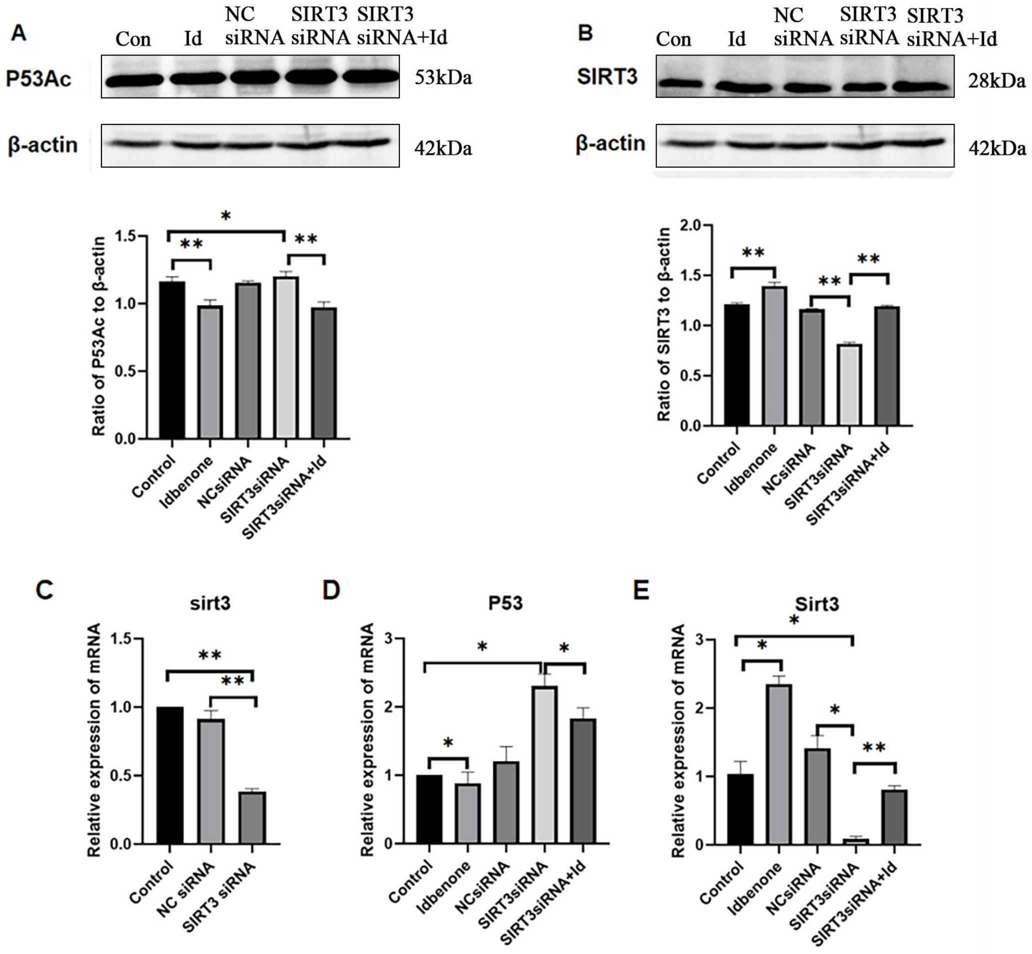
A
Con
Id
NC siRNA
SIRT3 siRNA
SIRT3 siRNA+Id
P53Ac
53kDa
β-actin
42kDa
Ratio of P53Ac to β-actin
Control
Idbenone
NCsiRNA
SIRT3siRNA
SIRT3siRNA+Id
B
Con
Id
NC siRNA
SIRT3 siRNA
SIRT3 siRNA+Id
SIRT3
28kDa
β-actin
42kDa
Ratio of SIRT3 to β-actin
Control
Idbenone
NCsiRNA
SIRT3siRNA
SIRT3siRNA+Id
C
sirt3
Relative expression of mRNA
Control
NC siRNA
SIRT3 siRNA
D
P53
Relative expression of mRNA
Control
Idbenone
NCsiRNA
SIRT3siRNA
SIRT3siRNA+Id
E
Sirt3
Relative expression of mRNA
Control
Idbenone
NCsiRNA
SIRT3siRNA
SIRT3siRNA+Id
